# Application value of high-frequency ultrasound combined with ultrasonography in the diagnosis of neonatal esophageal atresia

**DOI:** 10.4314/ahs.v23i3.63

**Published:** 2023-09

**Authors:** Yurong Ge, Baoyue Xu, Jing Shi, Weiwei Tang

**Affiliations:** 1 Department of Ultrasonic Medicine, Guiyang Maternal and Child Health Hospital, Guiyang, Guizhou P.R. China ,550003; 2 Weihai Municipal Hospital Shandong, China; 3 Department of Ultrasonic Medicine, Guiyang Maternal and Child Health Hospital, Guiyang, Guizhou, P.R. China, 550003; 4 Department of Ultrasound Medicine, Affiliated Hospital of Xiangnan University, Chenzhou, China

**Keywords:** High frequency ultrasound, Ultrasound angiography, Neonatal esophageal atresia, Application value

## Abstract

**Objective:**

To explore the application value of high-frequency ultrasound combined with ultrasonography in the diagnosis of neonatal esophageal atresia (EA)

**Methods:**

Seventy neonates with suspected EA who received healing in our hospital from August 2019 to April 2022 were retrospectively selected as the study subjects and their preoperative esophageal high-frequency ultrasound and ultrasound hydrography data were analysed. The diagnostic value of high-frequency ultrasound, ultrasound hydrography and combined detection in neonatal EA was analysed using intraoperative findings as the gold standard;

**Results:**

(1) Among the 70 children with suspected EA, 62 were confirmed to be positive and 8 were negative; 59 were positive and 11 were negative by ultrasound hydrography alone; 61 were positive and 9 were negative by high-frequency ultrasound alone; 62 were positive and 8 were negative by combined detection.

(2) The accuracy of combined detection was 97.14%, which was notably different from 92.86% by high-frequency ultrasound and 84.29% by ultrasound hydrography (P < 0.05).

(3) The diagnostic AUC of ultrasound hydrography, high-frequency ultrasound, and combined detection for EA was 0.6125, 0.6500, and 0.6563, respectively (P < 0.05).

(4) There was no significant variation in the distance between preoperative high-frequency ultrasound, ultrasound hydrography, and intraoperative measurements of distal and proximal blind ends of type IIIA and IIIB EA esophagus (P > 0.05);

**Conclusion:**

High frequency ultrasound and super fresh water injection angiography have good application value in the diagnosis of neonatal EA. There is no significant difference between the distance between the distal and proximal blind ends of the esophagus before and during the operation of type III EA and that during the operation of super fresh water injection angiography. However, in consideration of the risk of radiation and poisoning caused by esophagography, it is recommended that high-frequency ultrasound be selected first for diagnosis and if necessary, esophagography be supplemented for joint diagnosis.

## Introduction

Congenital esophageal atresia (EA) is a severe malformation of the digestive tract caused by abnormal development of the primary foregut that occurs during the embryonic period.^1^

Flow regulation shows that the incidence of esophageal atresia is approximately 1:2500 – 1:4500 2. The main cause of EA is esophagotracheal fistula caused by incomplete differentiation of esophagus and trachea. Most children with EA will be associated with organ malformation, which is difficult to treat. Studies have pointed out that EA is a complex disease involving multiple factors. Embryology points out that abnormal esophageal blood supply, abnormal local tissue differentiation and growth, or drug abuse, inflammatory stimulation, environmental pollution may be related to the occurrence and development of EA, but its specific mechanism still needs to be studied 3-4.

One-third of children with EA are premature infants, and their typical clinical symptoms include increased oral secretions, foaming at the mouth, or viscous vesicular secretions after birth, which may be associated with cyanosis, dyspnea, and even asphyxia 5.

Early differential diagnosis of EA is an important basis for the implementation of subsequent healing. The traditional EA identification method is esophageal iodized oil angiography, but this method has the risk of radiation exposure and contrast medium aspiration 6.

In recent years, with the continuous development of ultrasound technology, its advantages in the diagnosis and healing of EA have been continuously highlighted 7. This study intends to analyse the application value of high-frequency ultrasound and ultrasonic hydrography in the differentiation of EA in neonates by means of a controlled study in order to provide a reference for improving the prognosis of EA patients.

## Materials and methods

### General data

70 neonates with suspected EA who were healed in our hospital from August 2019 to April 2022 were retrospectively selected as the research subjects. There were 59 males and 21 females, with a median age of 7 hours to 5 days. age (3.10±0.89) d, birth weight 1.64kg-4.18kg, median body weight (2.74±0.56) kg; 20 of the 70 neonates were preterm infants (28.57%); The vital signs were stable and the typical clinical manifestations included foaming at the mouth, spitting up milk, cyanosis, difficulty in indwelling gastric tube, etc.; 32 of the 70 neonates were complicated with other malformations (8 situations of anal atresia complicated with vaginal fistula, 6 situations complicated with suburethral fistula, 5 situations were complicated by thoracic vertebra deformity, 3 situations were complicated by diaphragmatic bulging, and 10 situations were complicated by congenital heart disease).

### Inclusion criteria

1. All the enrolled children had clinical symptoms of suspected EA
2. The case data were complete
3. The investigation was reported to the hospital ethics committee for approval.

### Exclusion criteria

1. Children with concurrent pulmonary infection
2. Children who cannot tolerate EA surgery
3. Children who have been included in other clinical investigations.

### Intervention methods

The enrolled children underwent high-frequency ultrasound and ultrasound water injection contrast examination respectively.

The high-frequency ultrasonic testing application instrument is the Philips EPIQ5 ultrasonic instrument. The probe frequency is set to 5-12 MHz, and the esophagus and gastrointestinal tract of the children are scanned. During the testing, the children are placed in a supine position, their backs are raised, and the children's necks are fully exposed. The esophagus was scanned in multiple planes, directions, and angles at the front of the neck, parasternal and under the xiphoid process, and then the gastrointestinal tract was scanned, focusing on the continuity of the esophagus, the structure of the esophageal wall and the esophagus The inner diameter of the lumen is observed to check whether there is an esophagus-tracheal fistula in the child, and whether there is gas echo in the esophagus cavity and gastrointestinal tract of the child. heart malformation.

The ultrasonic water injection radiography was carried out by using a digital gastrointestinal X-ray machine. The head of the child was raised or the table was tilted so that the head of the child was high and the feet were low. A slightly hard No. 8 catheter was used to fill the contrast agent lipiodol into the esophagus. The head of the child was tilted back. Under television fluoroscopy, the mouth and nose were inserted into the esophagus until the front end of the catheter was blocked. Then 1-2ml lipiodol was injected. After the blind end of the esophagus was filled, the anteroposterior, left posterior oblique and right positions were taken for radiography respectively, the lipiodol contrast agent injected into the esophagus was immediately extracted.

The results of both imaging tests were blindly reviewed by 2 experienced radiologists and the diagnostic results were issued. If there was any disagreement, the assistance of a third physician could be sought.

### Observation indicators and evaluation standards

Using the intraoperative results of the enrolled children as the gold standard, the diagnostic efficacy of high-frequency ultrasound, ultrasonography, and combined detection for EA was calculated respectively; the diagnostic ROC curves of high-frequency ultrasound, ultrasonography, and combined detection for EA were drawn, and AUC was calculated. At the same time, the preoperative high-frequency ultrasound and ultrasonography were compared with the distance between the distal and proximal blind ends of type IIIA and IIIB esophagus measured during the operation.

### Statistical methods

T-test was used for the comparing of measurement data that obeyed normal distribution and homogeneity of variance, and was described by (mean ± standard deviation), and non-parametric data was used for skewed data or measurement data with unequal variance. The Mann-Whitney test (U test) in the test is described by the median (upper and bottom quartiles), and the measurement data is compared by the chi-square test, which is represented by the case (%), and the ROC curve is used to evaluate the diagnostic performance.

## Results

### Separate detection and combined detection and analysis of intraoperative results

**Figure 1:**
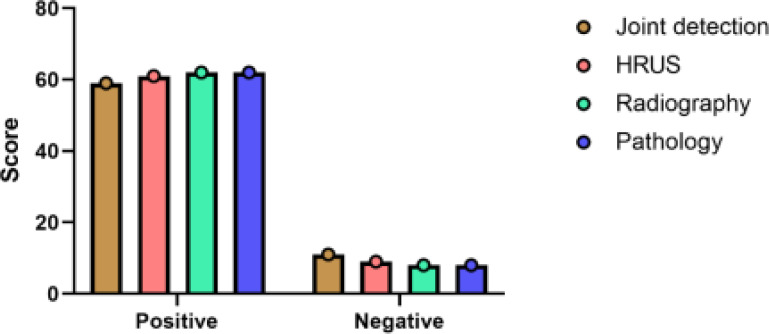
Individual detection, combined detection and analysis of intraoperative results

Taking the pathological test results as the gold standard, among the 70 children with suspected EA, 62 situations were confirmed positive and 8 situations were negative; 59 situations were positive and 11 situations were negative by ultrasound infusion alone; 61 situations were positive and 9 situations were negative by high-frequency ultrasound alone. Combined test was positive in 62 situations and negative in 8 situations. Table 1 and Figure 1.

**Table 1:** Analysis of single detection, combined detection and intraoperative results

Pathological findings	Ultrasound water injection angiography		High frequency ultrasound		Joint testing		Total
	
	Positive	Negative	Positive	Negative	Positive	Negative	
Positive	55	7	59	3	61	1	62
Negative	4	4	2	6	1	7	8
Total	59	11	61	9	62	8	70

### Comparing of diagnostic efficacy of single detection and combined detection

**Figure 2:**
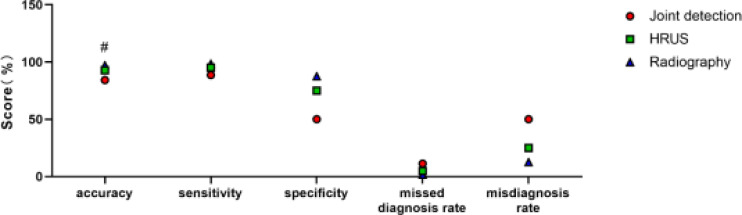
Comparing of the diagnostic efficacy of single detection and combined detection. The combined diagnostic accuracy was notably better than either single detection (P<0.05), # means that the variation between the same index groups was significant

Taking the intraoperative results as the gold standard, the diagnostic efficacy of contrast-enhanced ultrasound, high-frequency ultrasound and combined detection in EA were calculated and compared, and the results showed that the combined detection was notably better than either single detection in terms of diagnostic accuracy (P <0.05), Table 2 and Figure 2.

**Table 2:** Comparing of diagnostic efficacy of single detection and combined detection

Detection methods	Accuracy	Sensitivity	Specificity	Underdiagnosis rate	Misdiagnosis rate
Ultrasound water injection imaging	84.29% ( 59/70)	88.71% (55/62)	50.00% (4/8)	11.29% (7/62)	50.00% (4/8)
High frequency ultrasound	92.86% ( 65/70)	95.16% (59/62)	75.00% (6/8)	4.84% (3/62)	25.00% (2/8)
Combined detection	97.14% (68/70)	98.39% (61/62)	87.50% (7/8)	1.61% (1/62)	12.50% (1/8)
χ^2^	7.656	5.411	2.824	5.411	2.824
P	0.022	0.067	0.244	0.067	0.244

### ROC curve analysis of single detection and combined detection diagnosis

**Figure 3:**
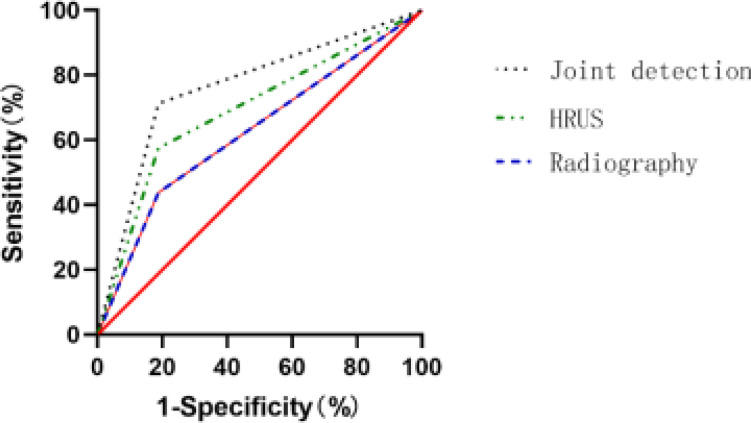
ROC curve of single detection and combined detection the diagnostic AUC of EA by contrast-enhanced ultrasound, high-frequency ultrasound, and combined detection were 0.6125, 0.6500, and 0.6563 (P<0.05)

The ROC curves for the diagnosis of EA by contrast-enhanced ultrasound, high-frequency ultrasound, and combined detection were drawn respectively, and the calculated AUCs were 0.6125, 0.6500, and 0.6563 (P<0.05), respectively, Table 3 and Figure 3.

**Table 3:** ROC curve analysis of single detection and combined detection diagnosis

Detection method	Sensitivity	Specificity	AUC	95% CI	SE	P
Ultrasound water injection imaging	72.09	77.78	0.6125	0.5251-0.6999	0.04461	0.0140
High frequency ultrasound	76.74	81.48	0.6500	0.5644-0.7356	0.04368	0.0011
Combined detection	95.35	92.59	0.6563	0.5710-0.7415	0.04349	0.0006

### Comparing of high-frequency ultrasound, ultrasonography, and intraoperative results

**Figure 4:**
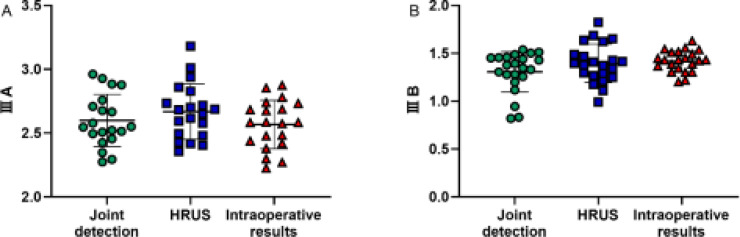
Comparing of high-frequency ultrasound, contrast-enhanced ultrasound and intraoperative results. There was no significant variation in the distance between the distal and proximal blind ends of type IIIA and IIIB esophagus between preoperative high-frequency ultrasound, contrast-enhanced ultrasound and intraoperative measurements (P> 0.05)

The comparing showed that there was no significant variation in the distance between the distal and proximal blind ends of the esophagus of type IIIA and IIIB between preoperative high-frequency ultrasound, ultrasonography and intraoperative measurement (P>0.05), Table 4 and Figure 4.

**Table 4:** Comparing of high-frequency ultrasound, ultrasonography, and intraoperative results

Detection method	Type IIIA (n=20)	Type III Bn=22)
Ultrasound water injection imaging	2.60±0.20	1.31±0.21
High frequency ultrasound	2.67±0.22	1.39±0.20
Intraoperative results	2.57±0.19	1.42±0.11
t	1.269	2.017
P	0.289	0.142

## Discussion

EA is an esophagus-tracheal fistula formed by the obstruction of esophageal cavitation at 3-6 weeks of embryonic development, resulting in incomplete separation of the esophagus and trachea.]. In the early 19th century, due to less research on EA and the lack of effective interventions, the mortality rate of EA was as high as 100%. It was not until the implementation of right extra pleural surgery and bilateral anastomosis in 1943 that the survival rate of children increased to 52%. % 10. In recent years, with the continuous progress of prenatal diagnosis technology, neonatal intensive care technology, and infant anesthesia technology, the cure situation of children with EA has been notably enhancedd. The cure rate of children with EA in some specialized healing centers at home and abroad can reach 95%. Above, only some children with severe respiratory distress, very low birth weight, or complicated deformities have poor prognosis 11-12.

Because the condition of children with EA is complex and most of them will be complicated by other deformities, it is of great guiding significance to determine the type of EA lesions before operation for the determination of subsequent healing plans 13. In this study, the differential diagnosis value of high-frequency ultrasound and water-infusion contrast-enhanced ultrasound in neonatal EA was analysed by means of comparative analysis. The results showed that the diagnostic sensitivity and specificity of water-infusion contrast-enhanced ultrasound for EA was 72.09% and 77.78%., the diagnostic sensitivity of high-frequency ultrasound for EA was 76.74%, and the specificity was 81.48%. Although it has good performance, it still has some shortcomings. A study of 24 patients with EA found that low-frequency and high-frequency color Doppler ultrasonography was performed on the patients respectively. 79.1%, 75.0%, and 79.1% of low-frequency ultrasonography had certain advantages 14. A study of 120 patients with EA found that the diagnostic accuracy rate of color Doppler ultrasonography for EA was 90.00%, the misdiagnosis rate was 3.57%, and the missed diagnosis rate was 6.25% 15. The above studies have certain similarities with this study.

The author of this paper analyses that ultrasound detection has the characteristics of convenience, non-invasiveness, and high tissue resolution. At the same time, it can also perform targeted scanning according to different high and low frequencies, thereby improving the accuracy of diagnosis. The esophagography implemented in this study is a traditional diagnostic measure for EA. Although it has certain diagnostic value, esophagography requires injection of a contrast agent, and patients often use the head down and feet high. The contrast agent easily enters the trachea through the epiglottis and is aspirated. There is also an increased risk of cancer in children with ionizing radiation 16. However, high-frequency ultrasound has the characteristics of good imaging effect. Because the thoracic and abdominal wall of the newborn is thinner, there is less gas in the lungs, and the ultrasound easily penetrates the thoracic and peritoneal membrane to clearly display the cervical and thoracic segments and the esophagus behind the heart, so there is no need to use contrast. Drugs and sedatives ae relatively safe 17. The results in this study show that high-frequency ultrasound has better diagnostic performance than water injection contrast-enhanced ultrasound, but it still has some shortcomings compared to combined detection. The misdiagnosis is mainly due to the wrong EA classification. The reasons for this phenomenon are as follows: (1) Under anesthesia, the blind end of the esophagus moves to a certain extent with breathing and swallowing activities, which affects the results 18; (2) Both sides When the blind end distance is about 2cm, it will have a certain impact on the judgment of EA subtype; (3) the operator's subjective factors. Therefore, joint detection can minimize the above errors and enhanced the diagnostic accuracy, and the results in this paper also confirm this point of view.

## Conclusion

High frequency ultrasound and super fresh water injection angiography have good application value in the diagnosis of neonatal EA. There is no significant difference between the distance between the distal and proximal blind ends of the esophagus before and during the operation of type III EA and that during the operation of super fresh water injection angiography. However, in consideration of the risk of radiation and poisoning caused by esophagography, it is recommended that high-frequency ultrasound be selected first for diagnosis, and if necessary, esophagography be supplemented for joint diagnosis.
